# Evaluating the Effectiveness of the SleepTracker App for Detecting Anxiety- and Depression-Related Sleep Disturbances

**DOI:** 10.3390/s24030722

**Published:** 2024-01-23

**Authors:** Doaa Alamoudi, Ian Nabney, Esther Crawley

**Affiliations:** 1 Department of Computer Science, University of Bristol, Bristol BS8 1UB, UK; ian.nabney@bristol.ac.uk; 2 Child Health, Bristol Medical School (PHS), University of Bristol, Bristol BS8 1UB, UK

**Keywords:** mHealth, insomnia, anxiety, depression, young adult

## Abstract

This study emphasises the critical role of quality sleep in physical and mental well-being, exploring its impact on bodily recovery and cognitive function. Investigating poor sleep quality in approximately 40% of individuals with insomnia symptoms, the research delves into its potential diagnostic relevance for depression and anxiety, with a focus on intervention in mental health by understanding sleep patterns, especially in young individuals. This study includes an exploration of phone usage habits among young adults during PPI sessions, providing insights for developing the SleepTracker app. This pivotal tool utilises phone usage and movement data from mobile device sensors to identify indicators of anxiety or depression, with participant information organised comprehensively in a table categorising condition related to phone usage and movement data. The analysis compares this data with survey results, incorporating scores from the Sleep Condition Indicator (SCI), Patient Health Questionnaire-9 (PHQ-9), and Generalised Anxiety Disorder-7 (GAD-7). Generated confusion matrices offer a detailed overview of the relationship between sleep metrics, phone usage, and movement data. In summary, this study reveals the accurate detection of negative sleep disruption instances by the classifier. However, improvements are needed in identifying positive instances, reflected in the F1-score of 0.5 and a precision result of 0.33. While early intervention potential is significant, this study emphasises the need for a larger participant pool to enhance the model’s performance.

## 1. Introduction

Sleep is a cornerstone of human health and well-being, intricately linked to various aspects of our overall wellness. Sleep is a natural state of the mind and body and occupies about one-quarter to one-third of the human lifespan. Before the 1950s, sleep was considered a passive practice that occurred as a result of a reduction in some vital force [1]. Now, it is understood that physiologically, sleep is the complicated process of repair and renewal for the body and mind. Scientists, however, do not have a conclusive explanation for why humans need sleep. We do remember that sleep is not a passive activity or a “switching off” of structure functions; sleep is thought to be valuable in some physiological operations, including the mental processing of experiences and the consolidation of memories [2].

This significance extends beyond humans, encompassing nearly all creatures. The five stages of sleep delineate this process. In Stage 1 (non-REM), the body transitions from wakefulness to sleep, lasting 1 to 7 min and comprising 2% to 5% of total sleep [3]. In Stage 2 (non-REM), a light sleep phase precedes deep sleep, lasting 10 to 25 min (45% to 55% of total sleep) [3]. During this stage, body temperature drops, heartbeat and breathing slow, and brain waves resist waking [4]. Stages 3 and 4 (non-REM) represent the deep sleep necessary for feeling refreshed, occurring in extended periods during the first third of the night. Here, eye movement, heartbeat, brain waves, and muscle activity reach their lowest levels [4]. Stage 3 lasts a few minutes (3–8% of sleep) [3], while Stage 4 lasts 20 to 40 min (10% to 15% of sleep) [3]. Lastly, in Stage 5 (REM), or rapid eye movement sleep, which commences about 90 min after falling asleep, the eyes move rapidly, brain waves resemble wakefulness, and heartbeat/breathing increase. Most dreaming occurs in this stage, and muscles become temporarily paralysed. Memory consolidation likely involves both REM and non-REM sleep [4].

Researchers classify sleep into two basic types: rapid eye movement (REM) sleep and non-REM sleep. Both types of sleep are linked to specific brain waves and neuronal activity [5]. During a typical night, individuals cycle through all stages of non-REM and REM sleep, with increasingly longer, deeper REM periods occurring toward morning.

Sleeping times are determined by two main factors: firstly, the body clock within our brain, which creates day–night rhythm cycles (circadian rhythm). The second factor is the chemical substances in our brain; the longer a person is awake, the more chemical pressure accumulates and the sleepier they feel [6]. Circadian rhythms result in individuals being categorised as “morning types” or “morning larks” (preferring early wake-up and nighttime sleep) or “evening types” or “night owls” (preferring daytime sleep and nighttime wakefulness). However, variations in sleep and wake-up times impact health, with sleep deprivation negatively affecting the brain, leading to lower alertness and energy levels [6].

Poor sleep quality has a profound impact on health, contributing to conditions such as anxiety, depression, heart disease, obesity, dementia, diabetes, and cancer [6,7]. Among university students, poor sleep quality is prevalent and is often intertwined with mental health challenges, heightening the risk of anxiety and depression [8,9]. Harvard Medical School’s Sleep Medicine Division has shed light on the sleep patterns of individuals facing stress, anxiety, and depression, revealing distinct characteristics such as lighter sleep, increased rapid eye movement (REM) sleep, and reduced deep sleep [10].

Sleep disorders, if untreated, can progress from acute insomnia to chronic insomnia. Moreover, poor sleep quality and mental health problems are common among university students, further increasing the risk of anxiety and depression [8,9]. The prevalence of anxiety and depression among young adults aged 16 to 24 in the UK is 18% to 21%, with higher rates among females at 22.5% compared to males at 16.8% [11,12].

Despite these challenges, young adults aged 18–35 experiencing depression and anxiety are less likely to start and complete treatment after referral by the National Health Service (NHS) to Improving Access to Psychological Therapies (IAPT) [13]. A significant percentage of these individuals do not engage with healthcare services, often due to lack of access [14].

Recognising the prevalence of mobile phone usage, with 58% of users downloading at least one health-related app, and the desire among outpatient psychiatry patients to use mental health apps [15,16], leveraging current technology could enhance accessibility to mental health services. Our research seeks to contribute to this domain by detecting changes in sleep patterns through phone usage (screen on/off) and accelerometer sensor data commonly found in smartphones. This sensor can detect alterations in the device’s position and orientation, aiding the understanding of users’ physical activity or restlessness during sleep.

We aim to influence public health interventions, ultimately reducing the prevalence of anxiety and depression among young adults. Through this refined narrative, we aspire to convey a clear, compelling, and well-justified argument that resonates with the broader scientific community.

## 2. Related Work

The advance of technology has enabled the employment of smartphone sensors by several groups to conduct sleep studies [17,18,19]. The accelerometer sensor, embedded in every smartphone, has been utilised to measure phone and body movement in order to monitor sleep stages [20]. Researchers have also analysed room environmental variables such as noise [10,20,21] and luminosity [10] in order to study their impact on sleep quality. The commonly used screen on/off timing in several studies has been shown to be a strong indicator of sleeping time and duration [20,22,23]. These sensors have been employed both individually and in combination as sleep detectors. While it is true that various factors, including diet, environmental conditions, and lifestyle, can contribute to insomnia, this study specifically concentrates on examining the association between insomnia and mental health issues, namely depression and anxiety. The focus on these two aspects allows for a more targeted investigation into the potential relationship between sleep disturbances and mental health well-being. While acknowledging the broader scope of potential insomnia causes, our emphasis on depression and anxiety aims to deepen the understanding of the specific mental health dimensions of sleep disruptions.

### 2.1. Single Modality Methods

The iSleep app [24], designed to replace wearable sleep trackers, utilises a smartphone’s built-in microphone to classify ambient noises and monitor sleep events. Despite achieving high accuracy in sleep event classification, the app necessitates daily user intervention, requiring specific room setups and expensive equipment. While successful in estimating sleep duration, its intricate procedure may prove challenging for daily implementation, especially for young adults. Additionally, the app’s testing on a specific age group may limit its generalizability, considering variations in phone usage across different age demographics.

Tappigraphy [25], a sensor measuring touchscreen events, shows promise in estimating sleep-onset and wake-up times comparable to actigraphy. However, it tends to consistently underestimate sleep duration due to variations in smartphone usage patterns. The study’s focus on a 24 h sleep–wake cycle might inaccurately reflect the diverse lifestyles of subjects, such as students or workers who may need to refrain from phone use during certain activities, impacting sleep time calculations.

Various mobile apps employing screen on/off events for sleep measurement, such as the “Know Addiction” app [19] and iSenseSleep app [23], show correlations between smartphone use and delayed sleep onset. However, limitations exist, including the predefined sleep window of 10 pm–10 am, potentially inaccurately estimating sleep duration. The iSenseSleep app, while providing estimates close to ground truth, is limited by its short study duration and may not capture changes in sleep patterns over time, especially in individuals dealing with mental health issues like depression or anxiety. Overall, these studies lack consideration of sleep quality and assume uniform sleep patterns for all users, neglecting individual preferences.

### 2.2. Multiple Sensors and Modalities

Z. Chen et al. [20], introduced “Best Effort Sleep” (BES), an Android app utilising various mobile sensors (light, microphone, phone usage, and stationary mode) to estimate sleep duration without user intervention. BES demonstrated a low standard error of ±42 min in sleep duration estimation over a week-long evaluation with eight participants. Despite its cost-effectiveness and minimal user involvement, relying solely on room environment observation may introduce inaccuracies, especially for individuals sleeping with lights on. Additionally, issues arise when users forget to recharge their phones, causing the app to mistakenly assume sleep during phone off mode. The study highlighted the significance of light and phone-off features in reducing errors.

“Toss ‘N’ Turn” (TNT) [17] is another Android app, aimed to detect bedtime, waketime, and sleep duration using multiple sensors, including the accelerometer, screen on/off events, light, microphone, and battery. The algorithm, running at 10 min intervals during normal sleep periods, achieved standard errors of ±35 min, ±31 min, and ±49 min for bedtime, waketime, and sleep duration, respectively. However, the app faced challenges when the mobile battery was low, causing data collection to stop.

Both BES and TNT studies emphasised the efficacy of accelerometer and screen events in predicting sleep time, while the light sensor’s predictability varied. Both studies utilised microphone sensors without addressing privacy concerns associated with collecting sensitive data from users’ smartphones.

While multiple-sensor approaches enhance sleep pattern understanding, the studies acknowledged the trade-off, with increased sensor usage potentially impacting battery life. The findings underscore the importance of optimising sensor selection to balance accuracy and energy efficiency in developing effective sleep monitoring apps.

Table 1 is an overall summary of methods used in the previous studies (Section 2.1 and Section 2.2) to determine sleep duration.

### 2.3. Smartphone Sensors for Detecting Depression and Anxiety

Recently, interest in studying the effectiveness of using smartphones for mental well-being have increased at a brisk pace. In order to link behavioural data to mental health, researchers have employed various software, smartphone sensors, and statistical approaches.

Geospatial activity (GPS) has been used to study mental health problems such as depression [27,28,29,30], bipolar disorder [31], stress [32,33], anxiety [32], and schizophrenia [28,29]. GPS alone cannot be considered a single intervention factor to determine mental health status. Depression or depressive symptoms could result from a chronic disorder or other dispositional factors that GPS cannot detect. Thus, identifying other factors of depression, such as sleep, may help provide early signals for depression symptoms. Also, the individual’s behaviour cannot be expected to be entirely consistent on a daily basis; even a severely ill person can have a good day. Another smartphone sensor, the microphone with voice recognition, has been employed in studies such as the Affective and Mental health MONitor (AMMON) and openSMILE libraries [34,35] to identify emotions and mood. Work on voice recognition is based on predefining several emotions and moods using audio and visual clips to create a library out of the collected voices. Yet, monitoring mental health through voice analysis is not enough, as there should be a reason for mood deterioration; a lack of sleep could be a factor that leads the individual to be in a bad mood during the day, which might influence an individual’s mood overall.

A considerable amount of literature has been published using mobile sensors to categorise mental health and well-being. The studies created platforms that used GPS, accelerometer, microphone, and phone usage sensors to detect individual mental health [33,36,37]. They discussed several mobile sensors and linked them to individual mental health and well-being.

The detailed literature in the field of tracking sleep using mobile sensors and detecting mental health problems using data collected from mobile sensors has been discussed and published in a narrative review paper [26]. The paper explores the potential of using smartphone sensors for tracking sleep patterns and detecting changes in mental health, specifically focusing on depression and anxiety. The review highlighted the prevalence of poor sleep and its impact on both physical and mental health. It emphasised the need for early monitoring and intervention to prevent severe mental health problems. The review involves an analysis of 12 articles that discuss the use of smartphone sensors to collect data on sleep patterns and mental health changes over time. Several technological methods investigated to detect sleep using smartphone sensors have linked changes in these sensors to mental health and well-being. The paper concludes by emphasising the potential of using smartphone sensors for unobtrusive data collection related to sleep patterns, depression, and anxiety. This presents a valuable research opportunity to detect insomnia early and provide timely interventions for mental health issues such as depression and anxiety.

Despite the limitations of these apps, the proliferation of digital technologies of mobile sensors can provide a feasible and unobtrusive method to continuously collect behavioural data from individuals, which can help in better understanding individual mental health conditions. Accelerometer and phone usage [27,33] features have proved their effectiveness in understanding an individual’s behaviour and mental well-being. Further study is needed in this area to understand the feasibility of using screen on/off events and accelerometer sensors to track sleep disorders and provide early intervention and treatment when insomnia is detected, in order to reduce mental health problems.

## 3. Study Design 

### 3.1. Patient and Public Involvement Group-PPI

According to Deloitte’s seventh annual mobile consumer survey (2019), around 79% of young adults check their phones before going to sleep [38]. Furthermore, 26% of the survey respondents answer messages even after falling asleep at night, while on the other hand, 89% of respondents use their phones within five to thirty minutes after they wake up. We conclude that technology use may be a useful medium to detect sleeping patterns and mental health deterioration before serious adverse events occur [38]. The contribution of this research is to develop a mobile app that can be used to collect passive data and can be used at a large scale as a public health intervention. The mobile app will be used to detect sleeping patterns and their relationship to anxiety and depression among young adults aged 18–25 years.

We held a virtual focus group with seven young people (five girls, two boys). We presented the topic “Mental health and its relations to sleep disorder” to the students and played a video showing how the app would run on their devices. Later, we ran a discussion session to obtain answers to the following questions:

- How do you usually wake up every day? Do you rely on an alarm? Or do you check your phone?
- What features do you think are important for young people to engage with the app?
- Do you think a silent app in the background is best? Or an app that you input into?
- Do you think young people will accept their data being collected?

The group indicated their preference for a user-friendly app that requires no user intervention and does not intrude on their privacy such as the use of the phone’s microphone or video camera. They were interested using an app that runs in the background and that helps them understand their sleeping patterns and mental health.

As for phone usage, most of the focus group members checked their phones before getting up from bed in the morning. They gave a mixed response regarding waking up to phone alarms and on their own.

### 3.2. The Development of the SleepTracker App

After an in-depth analysis of various literature sources, this led us to the development of the SleepTracker app to measure sleep disturbance. In this context, night waking is identified as an instance when the screen is activated or when movement is detected during the night. The frequency of these occurrences serves as a metric to ascertain the presence of sleep disturbance.

It is essential to emphasise that the exact testing of the SleepTracker app was exclusively carried out on Android devices using Android Studio as a programming language to develop the app. This decision was prompted by specific limitations imposed by iOS, particularly its constraints on background app functionality. The core purpose of the SleepTracker app was conceived and executed as a background process on users’ devices upon user consent. Unfortunately, this service is not available on iOS devices. Our discussions with an Apple representative solidified the fact that the capability to operate an app in the background is restricted by the company’s policies. Consequently, iOS users are compelled to manually launch the app each time prior to commencing their sleep routine. The other challenge with iOS is over data ownership. The utilisation of Apple HealthKit entails the mandatory submission of all health-related data to Apple. However, this process does not grant us ownership or comprehensive control over our own data [39].

Conversely, Android provides a service that seamlessly aligns with the requisites of our study. In our implementation, we utilised a “foreground service” [40] for this purpose. The primary function of this service is employed for tasks that demand user awareness. For instance, an audio application would leverage a foreground service to play audio content. Foreground services necessitate the presentation of a notification. These services persist even when user interaction with the app is minimal [40].

When employing a foreground service, it is obligatory to display a notification, ensuring users are actively informed about the ongoing service operation. This notification remains unremovable unless the service is either terminated or withdrawn from its foreground state [40]. This same approach was incorporated in the SleepTracker app, where a notification informing the user of the app running in the background was consistently displayed each time the app commenced its operation. Here is how the implementation works:

1. User Initiation: When a user signs up for the app and provides the required information, including email, age, gender, and their preferred sleep time window (start and end times), the SleepTracker app initiates its core functionality. This functionality operates within the specified sleep time window, involving the collection of sleep-related data and their synchronization with the AWS backend database.
2. Starting the Foreground Service: Upon starting its core functionality, the SleepTracker app initiates a foreground service. The foreground service is a component that runs independently in the background but is elevated in priority, meaning it is less likely to be killed by the system to free up resources.
3. Displaying a Persistent Notification: As part of using a foreground service, the SleepTracker app displays a persistent notification to inform the user that the app is actively running in the background during the sleeping window. This notification is crucial for transparency and compliance with Android’s guidelines. The notification typically contains relevant information about the app’s operation, such as “SleepTracker is running,” along with the app’s icon.
  As stated, the notification displayed by the foreground service remains unremovable by the user. This is a safeguard against malicious apps that might try to run services in the background without the user’s knowledge. In the case of the SleepTracker app, the notification remains visible to the user until either the service is terminated, or the app is withdrawn from its foreground state.
4. Real-time Data Synchronization: The app is linked to a secure online database implemented using AWS. The database supports the synchronization of data in real-time whilst the app is still running. It also provides security for the collected data and ensures that authentication occurs before accessing the database. The privacy policy by Amazon does not allow its employees access to our data. AWS protects data from unauthorised access by encrypting data while stored. AWS encrypts an app’s build artifacts by default, using AWS KMS keys for Amazon S3 that are managed by the AWS Key Management Service. In order to reduce the likelihood of accessing the participant’s data, we created two separate databases:
  - Database 1: To store personal information such as name, email, age, and ethnicity.
  - Database 2: To store data collected from the mobile sensors and survey score.
  To increase the granularity of the data, a range of data has strict rules that cannot be modified or updated once it is submitted. The data stored in Database 1 do not permit the user to update or modify. In Database 2, data related to the SleepTracker app, the users can only read the daily data about sleep duration and total movements during sleep duration. The only data modification or update permissible to the user is their sleep and wake-up times.
5. User Setup–Inputting Vital Information: Following installation, users are guided through inputting essential information such as email addresses, gender, and age. In a bid to enhance battery efficiency, we kindly asked users to enter their sleep onset and wake-up times. This cooperative approach enables the app to function within predetermined windows, effectively conserving battery life.
6. Data Extraction for Comprehensive Analysis: Upon the study’s culmination, we extract the accumulated data into an excel file, ready for thorough analysis. This dataset comprehensively captures all interactions with the phone, encompassing screen on/off events and accelerometer sensors.

## 4. Recruitment

In this comprehensive study, we extended invitations to students aged 18–25 from the University of Bristol who experienced difficulties falling asleep, restlessness, or frequent sleep disturbances. Our recruitment strategy aimed to assemble a participant cohort with specific sleep-related challenges. To fortify the robustness and integrity of our research, we implemented exclusion criteria, meticulously refining our participant pool. Individuals failing to meet any of the following criteria were excluded from the study: those who utilised the app for less than 28 days, individuals outside the specified age range of 18 to 25, non-students at the University of Bristol, those not utilising an Android smartphone, and individuals with a history of, or undergoing treatment for, anxiety or depression. These exclusion criteria were thoughtfully selected to ensure a more focused and homogeneous participant group, aligning closely with the objectives of our research. By adhering to these criteria, we aimed to contribute to the overall quality and reliability of our study outcomes. These carefully established criteria served to enhance the coherence and relevance of our findings, maintaining a sharp focus on the intended scope of our research. Through this approach, we strove to elevate the overall quality of our study and derive meaningful insights into the sleep patterns and challenges faced by young adults within the University of Bristol community.

To ensure a diverse and comprehensive sample, the recruitment drive for this study was conducted over a period of six months, from October 2022 to April 2023.

The aim of the recruitment drive was to reach a large number of potential participants and increase the chances of recruiting a representative sample. In order to achieve this, a variety of recruitment methods were utilised, including the distribution of posters in and around university buildings and targeted advertisements on Facebook.

The Facebook advertisements were specifically designed to reach the students at the University of Bristol, ensuring that the study was promoted to the appropriate audience. By utilising a range of recruitment methods, the study team was able to engage with a broad cross-section of the university population, increasing the likelihood of recruiting a diverse sample.

### Demographic Samples

All participants were university students aged 18–25. Table 2 is a summary of the participants’ gender and ethnicity. The study excluded participants who met the following criteria: those who used the app for less than 28 days, individuals outside the age range of 18 to 25, non-students at the University of Bristol, those not using an Android smartphone, and individuals with a history of, or currently receiving treatment for, anxiety or depression.

## 5. Instruments

### Detecting Sleep Disturbance and Mental Health Problems

**Figure 1:**
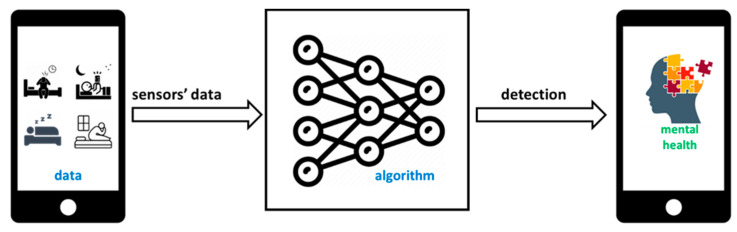
Overview of the SleepTracker app.

By collecting data on users’ sleeping patterns, we hoped to gain insights into the relationship between sleep disturbance, depression, anxiety, and the potential of the SleepTracker app to intervene and help individuals identify and manage these symptoms. Figure 1 shows the overview of the study objective. A screenshot showcasing the user experience can be found in Appendix A.

In this study, sleep disturbance was measured by using the screen on/off events and accelerometer sensors. The app defined a diurnal rhythm for which the users recorded their sleeping window (i.e., the times that they normally sleep). We used this method because individuals had different sleep-time windows; therefore, the app enabled them to specify the earliest time they went to bed and the latest time they woke up in the morning. This method also helped to reduce battery usage by having the app remain inactive except during sleeping time. This field test aimed to compare the actual sleeping time recorded in a sleep diary with the corresponding time measured by the app.

The app was designed to prompt the users to enter their sleep/wake-up time window. Allowing for the fact that users might oversleep, the app continued collecting data beyond the wake-up time window entered by the user if the screen was off. In instances where the screen activated briefly during the night, lasting less than five minutes, the algorithm adopted the preceding screen-off event, a methodology validated in previous testing within iSenseSleep [23]. The app operated in the background, monitoring the phone’s accelerometer sensor and screen on/off events, with data recorded on a remote database. An algorithm was employed to identify sleep disturbance, defined as follows:

- A total of 3 periods of phone usage over 5 min per night, for 5 nights out of 7;
- A total of 3 periods of >20 phone movements within 30 min per night, for 5 nights out of 7.

This algorithm served as a valuable tool for identifying individuals experiencing insomnia, particularly those struggling to maintain uninterrupted sleep with frequent wake-up events during the night—an indicative symptom of acute insomnia. Acute insomnia, characterised by difficulties in both falling and staying asleep, leads to compromised sleep quality and typically persists for a brief duration, ranging from a few nights to a few weeks. Various factors, including stress, anxiety, environmental changes, or specific life events, can contribute to the onset of acute insomnia [41].

In terms of screen on/off events, our algorithm identified sleep disturbance when users engaged in three or more periods of phone usage lasting over five minutes each night, observed on five out of seven nights. Additionally, concerning phone movements, the algorithm assessed each night for three instances of more than 20 phone movements occurring within a 30 min timeframe, observed on five out of seven nights. In our research, we utilised the accelerometer sensor to track phone movements, setting a threshold of 20 movements based on insights from a previous study [42]. The analysis of movement data, when juxtaposed with users’ sleep diaries, revealed a discernible pattern: individuals who were awake consistently exhibited a higher frequency of phone movements compared to those who were asleep.

On Professor Crawley’s advice, the app was programmed to send notifications to users to take a survey if sleep disturbances were detected over five consecutive nights. We chose to send a notification over a seven-night threshold to the user to check whether the app’s detection of a sleep disturbance corresponded with the user’s experience. According to the National Institute for Health and Care Excellence (NICE) guidelines, it is recommended to employ routine outcome measures for individuals exhibiting symptoms of anxiety or depression. Additionally, it is advised to conduct further assessments at intervals of no more than two weeks [43]. In addition, according to the Nuffield Trust for the Treatment of Patients with Major Depressive Disorder, a follow-up visit within 7 to 14 days after initiating treatment with antidepressant medication [44] is recommended.

To track changes in the users’ mood and mental health, the SleepTracker app prompts the user to complete three questionnaires on the app. The first is the Sleep Condition Indicator (SCI) [45], a self-rating scale that assesses insomnia disorder based on the Diagnostic and Statistical Manual of Mental Disorders (DSM-5) criteria. The SCI scale ranges from 0 to 10, with higher scores indicating better sleep while scores below five are considered a sign of sleep disorder. The second questionnaire is the Generalised Anxiety Disorder-7 (GAD-7) [46], and the third is the Patient Health Questionnaire-9 (PHQ-9) [47]. A score of more than 9 in either GAD-7 or PHQ-9 questionnaires indicates symptoms of anxiety and/or depression. These are screening methods used to detect insomnia, generalised anxiety, and depression, respectively. The measurement tools employed in our study, Sleep Condition Indicator (SCI), Patient Health Questionnaire-9 (PHQ-9), and Generalised Anxiety Disorder-7 (GAD-7), has been rigorously assessed [45,48,49]. Extensive validation studies support the reliability and accuracy of these instruments in individually measuring sleep conditions, depressive symptoms, and generalised anxiety. The use of well-established and validated tools ensures the credibility and robustness of our data, reinforcing the validity of the measurements and the overall integrity of our research findings.

If the app detected any specified criteria indicative of sleep disturbances, it promptly notified the user. This notification communicated our observation from the past 7 days, emphasising identified sleep disturbances and suggesting a potential link to difficulties in sleep. Users could easily click on the notification to participate in a survey assessing problems related to anxiety or depression. The resulting score seamlessly recorded within the app, allowing users to complete the questionnaires at their convenience.

Upon questionnaire completion, the criteria reset, and the app continued monitoring sleep patterns. Throughout the study, participants completed questionnaires at 28 and 56 days. The maximum frequency for completing questionnaires was five times (initial download at the 28th and 56th day, and once monthly if issues were detected). Most participants only completed the questionnaires three times during the 56-day period. This streamlined process ensured timely intervention and continuous monitoring while offering users flexibility in survey participation.

A score of more than 9 on either the GAD-7 or PHQ-9 scales indicates that symptoms of moderate anxiety/depression are present. If a participant scored more than 9 at any point in the study, they would not be asked to complete any more questionnaires until the final one at the end of the 56-day study period. We continued to track their sleep habits, provided they did not withdraw from participation. We also signposted to relevant online resources and advice on seeking help from Student Health.

## 6. Data Analysis Procedures

The data analysis process for our SleepTracker study was comprehensive and involved multiple key steps. Initially, we collected data from the SleepTracker app, specifically focusing on sleep disturbance calculations derived from phone usage and movement data. To ensure data quality, we addressed missing values by replacing them with the previous day’s data.

To generate the matrices presented in the results section, we initiated the process by constructing a comprehensive table encompassing the two distinct conditions identified by the SleepTracker app. These conditions included Screen on/off events occurring three times per night for five consecutive days and phone movements happening three times per night for five consecutive days. The ensuing table outlines the instances of sleep disturbances identified by the app for each user. A summary of the table can be found in Appendix B.

Subsequently, we provided a comparative analysis of survey scores (e.g., SCI, GAD-7, and PHQ-9) obtained at the commencement, midpoint, and conclusion of the study. Participants were required to complete the survey a minimum of two times and a maximum of four times throughout the study period. Our examination involved contrasting the two conditions of sleep disturbances detected by the app with the corresponding survey scores.

Specifically, the Sleep Condition Indicator (SCI) score was derived from the cumulative values of individual items, covering a scale from 0 to 10. A higher overall SCI score indicated an enhancement in sleep quality. By contrast, for both PHQ-9 and GAD-7, participants scoring below 9 were considered within the normal range. This comparative analysis aimed to unveil potential correlations between app-detected sleep disturbances and survey scores, offering insights into the relationship between sleep patterns and psychological well-being.

This structured approach allows us to derive nuanced insights into the impact of phone usage and movements on sleep-related indicators, offering a robust foundation for drawing meaningful conclusions from our SleepTracker study.

## 7. Results

### 7.1. Relationship between Sleep Disturbance Detected by the SleepTracker App and SCI Results

In a 56-day study with 11 participants, we evaluated a model’s performance in detecting sleep disturbances using the Sleep Condition Indicator (SCI) score and a confusion matrix. The SCI score, ranging from 0 to 10, is derived from participants’ responses to questions, with higher scores indicating better sleep quality. The model accurately identified sleep disturbances in 3 out of 11 users with low SCI scores (true positives), avoiding false negatives. However, it exhibited a notable number of false positives, wrongly identifying sleep disturbances in 6 users without low SCI scores. Given the small sample size, expanding participant numbers is crucial to establish the model’s effectiveness in identifying sleep disturbances more reliably and reducing false positives. In this study, individuals with a sleep disturbance score equal to 1 were excluded from the positive detection category. Instead, we incorporated them into the true negative classification. The results are presented in Table 3.

### 7.2. Relationship between Sleep Disturbance Detected by the SleepTracker App and Depression and Anxiety

In examining the link between sleep disturbance, anxiety, and depression, we analysed participants’ questionnaire responses, comparing them with sleep disturbance indicators. Scores below 9 were considered normal. Table 4 illustrates this relationship, presenting insights into the model’s performance in predicting sleep disturbances based on PHQ-9 questionnaire responses. Notably, the model accurately identified sleep disturbances in 3 true positive cases, correctly indicating “Yes” for participants with PHQ-9 scores of 9 or higher, and showed no false negatives, indicating it did not miss any sleep disturbances among participants with elevated PHQ-9 scores.

Table 5 delves into the correlation between sleep disturbance and GAD-7 scores, showcasing the model’s proficiency in identifying true positives and true negatives based on participants’ GAD-7 scores. While effectively recognising sleep disturbances in 3 out of 9 participants with elevated GAD-7 scores (true positives) and showing no false negatives, the model raises concern with false positives, implying potential over-sensitivity and the risk of unwarranted alarms.

From the preceding tables, we can derive class-wise accuracy, precision, recall, and F1-scores, consolidating the outcomes in Table 6. Recall, a pivotal metric, gauges the ratio of correctly predicted positive observations to the total actual positives, thereby assessing the model’s proficiency in capturing all relevant instances within the dataset. Accuracy, a comprehensive metric, represents the ratio of correctly predicted observations to the total observations, offering a holistic measure of the model’s overall performance. Precision, an indicator of positive prediction accuracy, delineates the ratio of correctly predicted positive observations to the total predicted positives. Specificity, a measure of the model’s capability to avoid false alarms for the negative class, is expressed as the ratio of correctly predicted negative observations to the total actual negatives. The F1-Score, an amalgamation of precision and recall through harmonic mean, emerges as a balanced metric, particularly beneficial in scenarios featuring an uneven class distribution. These metrics form integral components of the evaluation process for machine learning models, particularly in instances where class distributions may be imbalanced. Each metric contributes a unique perspective on the model’s performance, chosen based on the distinct goals and requisites of the classification task.

Table 6 shows the error metric measuring model performance by calculating the mean of sensitivity and specificity. The result of the F1-Score indicates that there is a relationship between sleep disturbance algorithm with the SCI, PHQ-9, and GAD-7 scores. However, an F1-Score of 50% indicates a moderately good performance and that there is room for improvement in the algorithm.

All three models exhibited a perfect recall score of 1.00 (100%). This signifies their strong capability to accurately identify instances of “Sleep Disturbance” (true positives) among all actual “Sleep Disturbance” cases. However, while the recall score was high, the accuracy score for all three models was 0.45 (45%), implying that only 45% of the model’s predictions were correct. This suggests a notable number of false positive predictions, where negative cases are incorrectly classified as positive.

Additionally, the precision score was 33%, indicating that one-third of the instances predicted as “Sleep Disturbance” were indeed “Sleep Disturbance,” while the remaining two-thirds were false positives. Furthermore, the specificity measures were at 25%, indicating that the models struggled to accurately predict “non-Sleep Disturbance” cases.

## 8. Discussion

In the results section, we delved into the association between sleep disturbances and SCI, PHQ-9, and GAD-7 scores. The analysis of the confusion matrix in Table 5, depicting the performance of the proposed algorithm, showcases an impressive recall level, attaining a perfect score of 1.00. This indicates the model’s adeptness in accurately identifying “Sleep Disturbance” cases among all instances of actual “Sleep Disturbance.”

Despite this commendable recall, the overall accuracy of the model stands at 45%, primarily influenced by a notable count of false positive predictions. These instances involve non-Sleep Disturbance cases being mistakenly classified as sleep disturbances. The precision score, at 33%, underscores that only one-third of the predicted sleep disturbance instances are true positives, leaving the remaining two-thirds as false positives.

Furthermore, the low specificity measures of 25% indicate that the model encounters challenges in precisely identifying cases with no sleep disturbance. In summary, while the model demonstrates proficiency in recognising genuine sleep disturbance cases, there exists an opportunity for enhancement by reducing false positives and improving specificity for instances with no sleep disturbance.

## 9. Challenges and Recommendations

A key challenge emerged regarding the number of participants. Due to a limited participant pool, we acknowledged the need for further studies to ensure broader applicability and robustness of the findings. Statistical power analysis indicates that, in order to attain a power of 0.95 while upholding a significance level of 0.05, a requisite sample size of 134 participants is crucial. This size is essential for detecting a point biserial correlation of 0.30 between a continuous variable and a binary outcome.

In advancing the field of sleep-tracking technology, to strengthen the robustness and generalizability of the findings, it is recommended to conduct studies with a larger and more diverse sample size. This expansion would offer comprehensive insights into the effects of sleep monitoring apps like SleepTracker on mental health, reducing the risk of false negative findings. Additionally, future research endeavours should focus on refining algorithms to better measure sleep disturbance thresholds. This includes exploring algorithms that specifically account for phone usage periods lasting over 5 min or involving more than 20 phone movements within 30 min per night for at least 5 nights out of 7. Customised algorithms for these patterns could provide valuable insights into the relationship between phone usage and sleep disruptions. Furthermore, this study suggests incorporating clinicians into the technology to enhance the app’s performance and contribute to treatment decisions related to sleep disturbances. Finally, future research could delve into mental health interventions, exploring real-time symptom-monitoring systems and behavioural changes that signal deteriorating mental health states, ultimately working towards improving overall mental health and well-being.

## 10. Conclusions

This paper has discussed the use of mobile sensors to predict mental health problems. The SleepTracker app was developed and tested to measure insomnia and its relationship to depression and anxiety. The app is equipped to detect insomnia at an early stage. In order to improve the accuracy of predicting insomnia symptoms, the SleepTracker app needs to increase the threshold of sleep disturbance of phone movements. However, this study acknowledges the shortage in the number of the participants, which renders the performance model sans perfect results.

In summary, the findings of this study suggest that mobile phone sensors can be used to predict mental health problems that influence sleeping patterns, but further research is needed to optimise its accuracy and validate its effectiveness.

In this study, our primary focus was on students at the University of Bristol. Concurrently, we are actively engaged in a broader project that extends its scope to school-age children, encompassing a more extensive participant pool.

## Figures and Tables

**Table 1:** Studies of mobile sensors to monitor sleep.

Author(s)	Year	Sample Size	Study Length (Days)	Accelerometer	Screen on/off Event	Light Sensor	Stationary Mode	Microphone	Battery	Touch Screen Event
T. Hao et al. [24]	2013	7	51					√ ^a^		
J.N Borger et al. [25]	2019	79	1400							√
S.Abdullah et al. [22]	2014	9	97		√					
Y.H Lin et al. [19]	2019	61	14		√					
M.Ciman et al. [23]	2019	14	180		√					
Z.Chen et al. [20]	2016	8	7		√	√	√	√		
J.K Min et al. [17]	2014	27	30	√	√	√		√	√	

^a^√: indicates the type of sensor used in the study. Source: The Feasibility of Using Smartphone Sensors to Track Insomnia, Depression, and Anxiety in Adults and Young Adults: Narrative Review (Doaa Alamoudi*, Emma Breeze, Esther M. Crawley, Ian Nabney) [26].

**Table 2:** Summary of the sample size and their ethnicity.

Demographic Category	Frequency
Gender	
Male	4
Female	7
Ethnicity	
White (including any white ethnic group)	6
Other ethnic group	5

**Table 3:** Sleep disturbance vs. SCI score.

			Predicted Classification		
			Sleep Disturbance		
			Yes	No	Total
**Actual classification**	**SCI ≤ 5**	**Yes**	TP = 3	FN = 0	3
		**No**	FP = 6	TN = 2	8
		**Total**	9	2	11

**Table 4:** Sleep disturbance vs. PHQ-9 score.

			Predicted Classification		
			Sleep Disturbance		
			Yes	No	Total
**Actual classification**	**PHQ-9 ≥ 9**	**Yes**	TP = 3	FN = 0	3
		**No**	FP = 6	TN = 2	8
		**Total**	9	2	11

**Table 5:** Sleep disturbance vs. GAD-7 score.

			Predicted Classification		
			Sleep Disturbance		
			Yes	No	Total
**Actual classification**	**GAD-7 ≥ 9**	**Yes**	TP = 3	FN = 0	3
		**No**	FP = 6	TN = 2	8
		**Total**	9	2	11

**Table 6:** Model performance.

Sleep Disturbance vs.	Recall	Accuracy	Precision	Specificity	F1-Score
SCI score	1.00	0.45	0.33	0.25	0.50
PHQ-9 score	1.00	0.45	0.33	0.25	0.50
GAD-7 score	1.00	0.45	0.33	0.25	0.50

**Figure A1:**
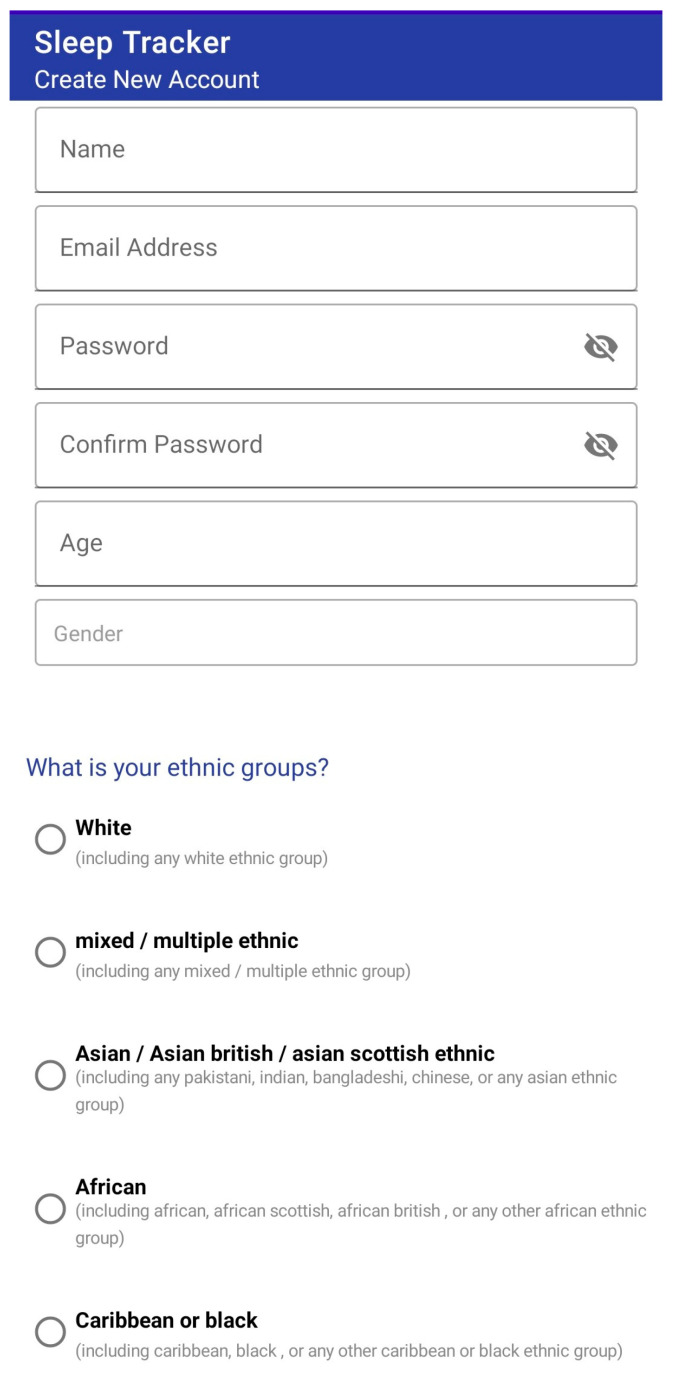

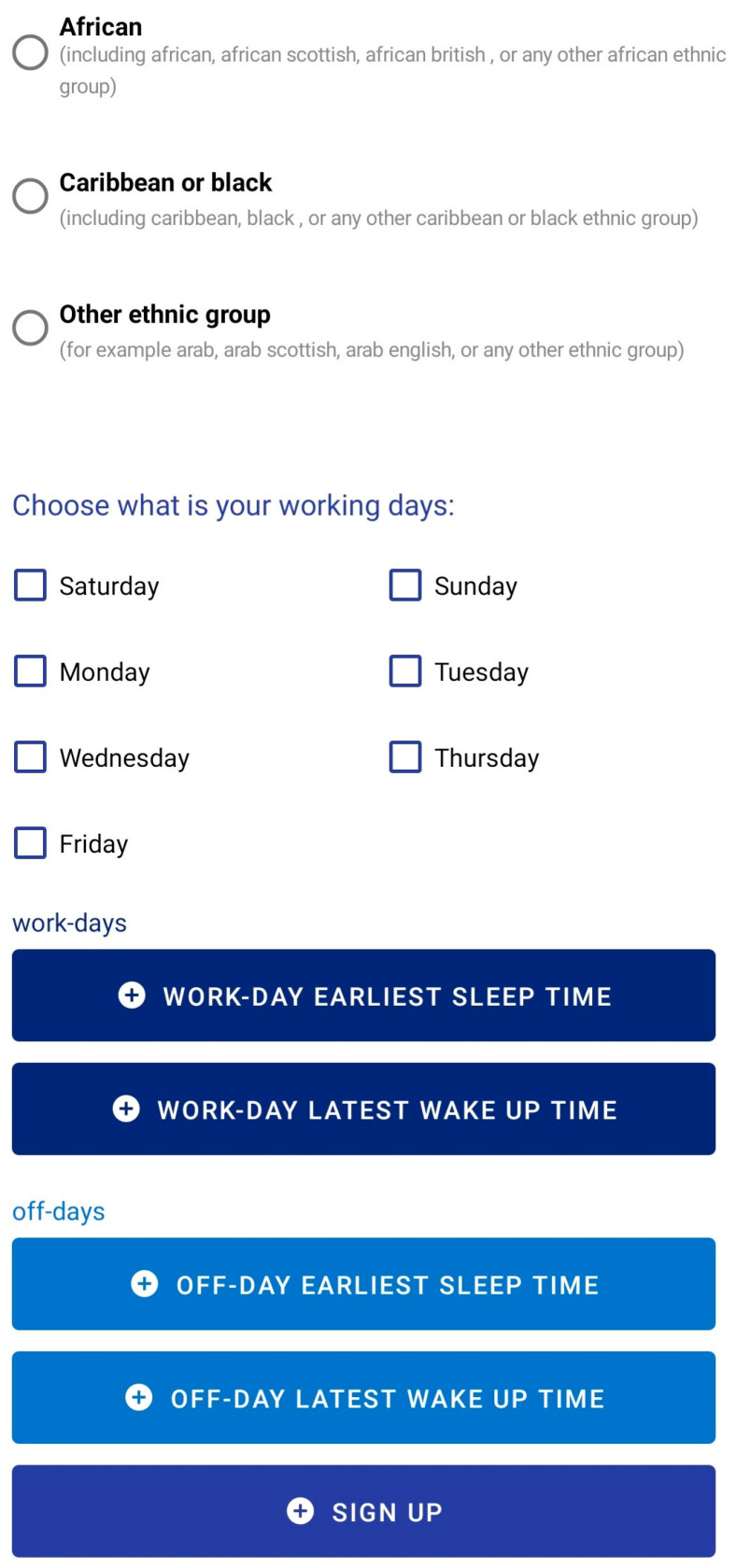
Sign-up page.

**Figure A2:**
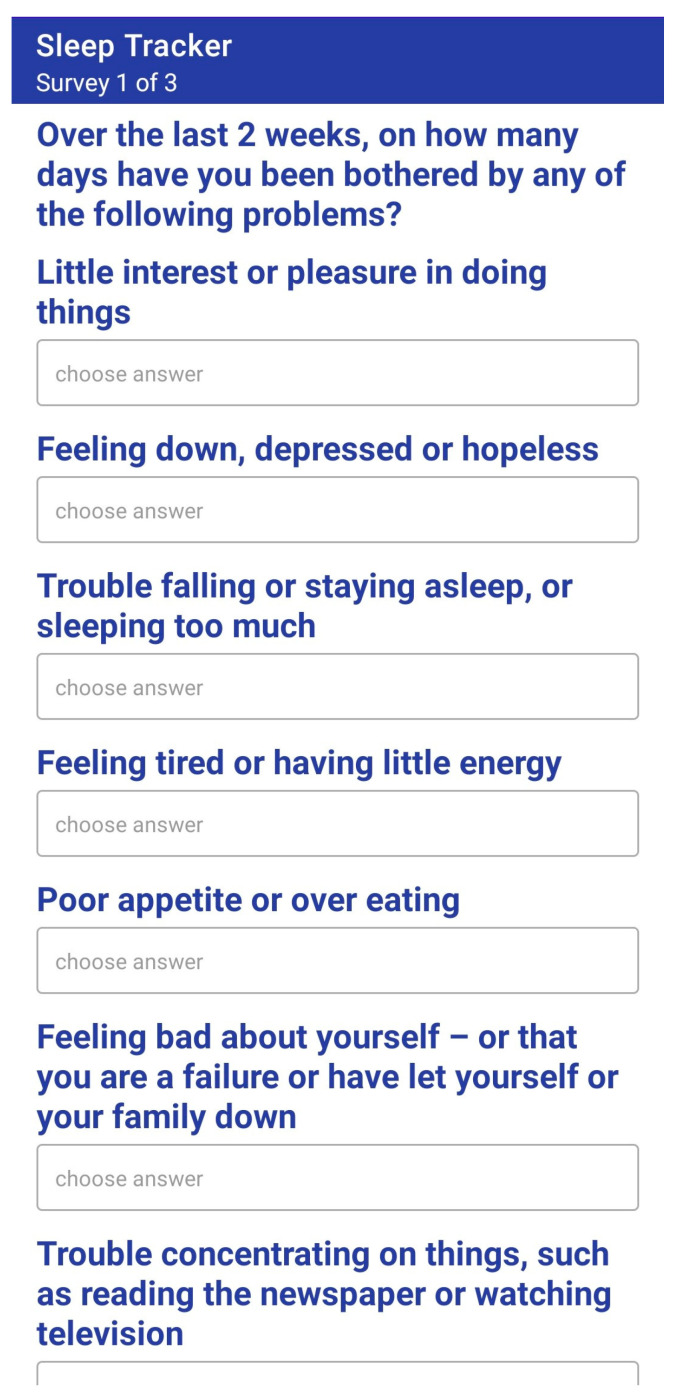
Survey page.

**Figure A3:**
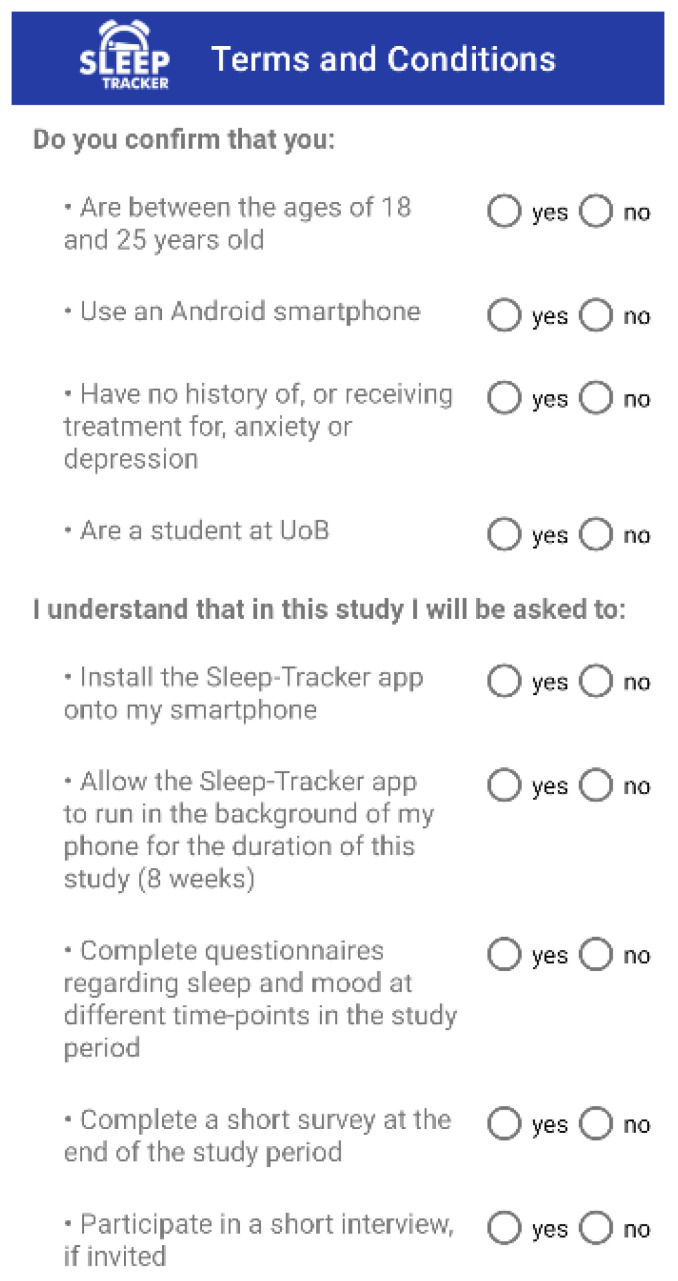
Terms and conditions.

**Figure A4:**
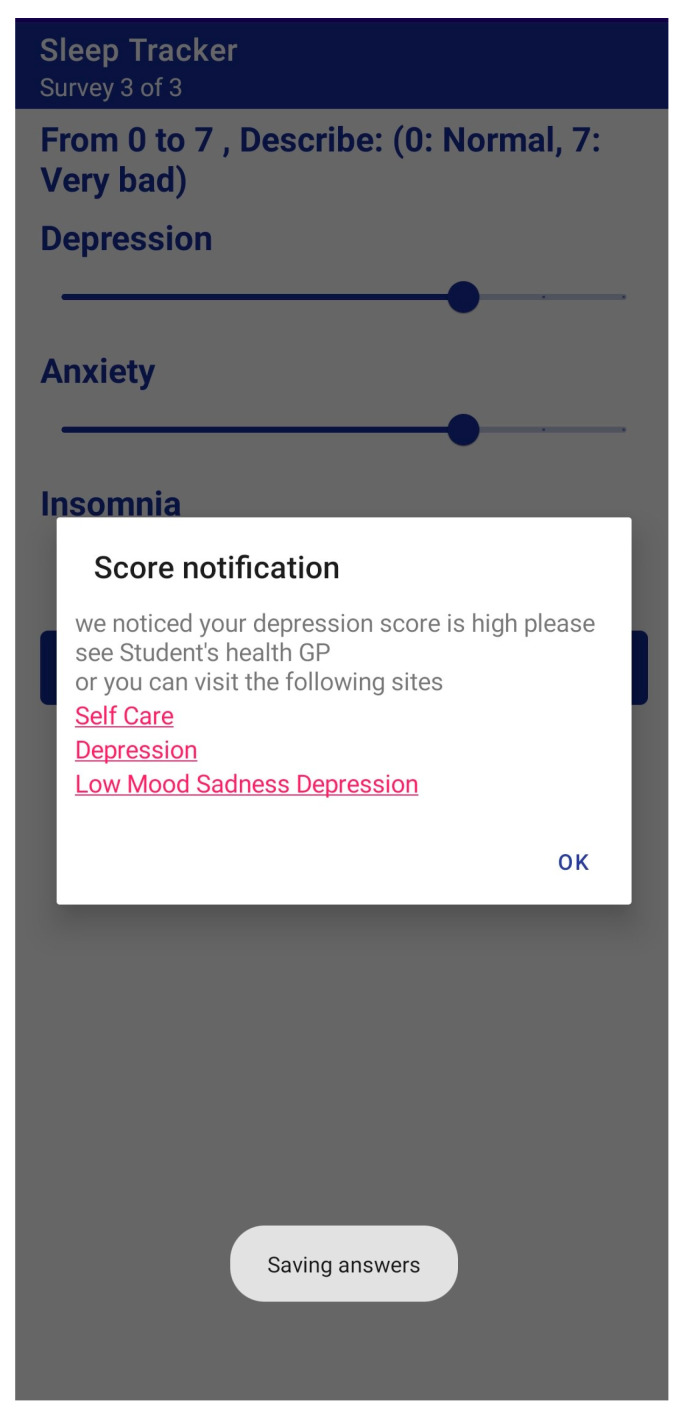
A notification if the depression or anxiety score was more than 9.

**Figure A5:**
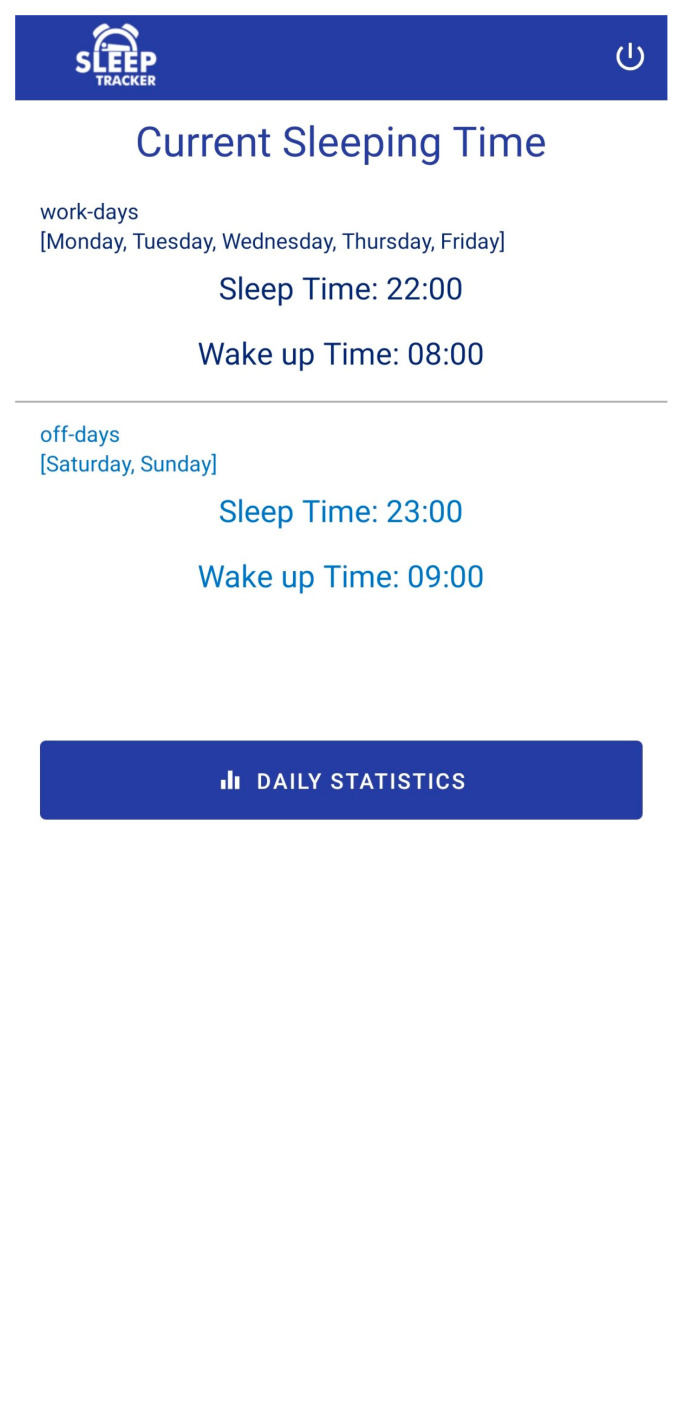
Home page.

**Figure A6:**
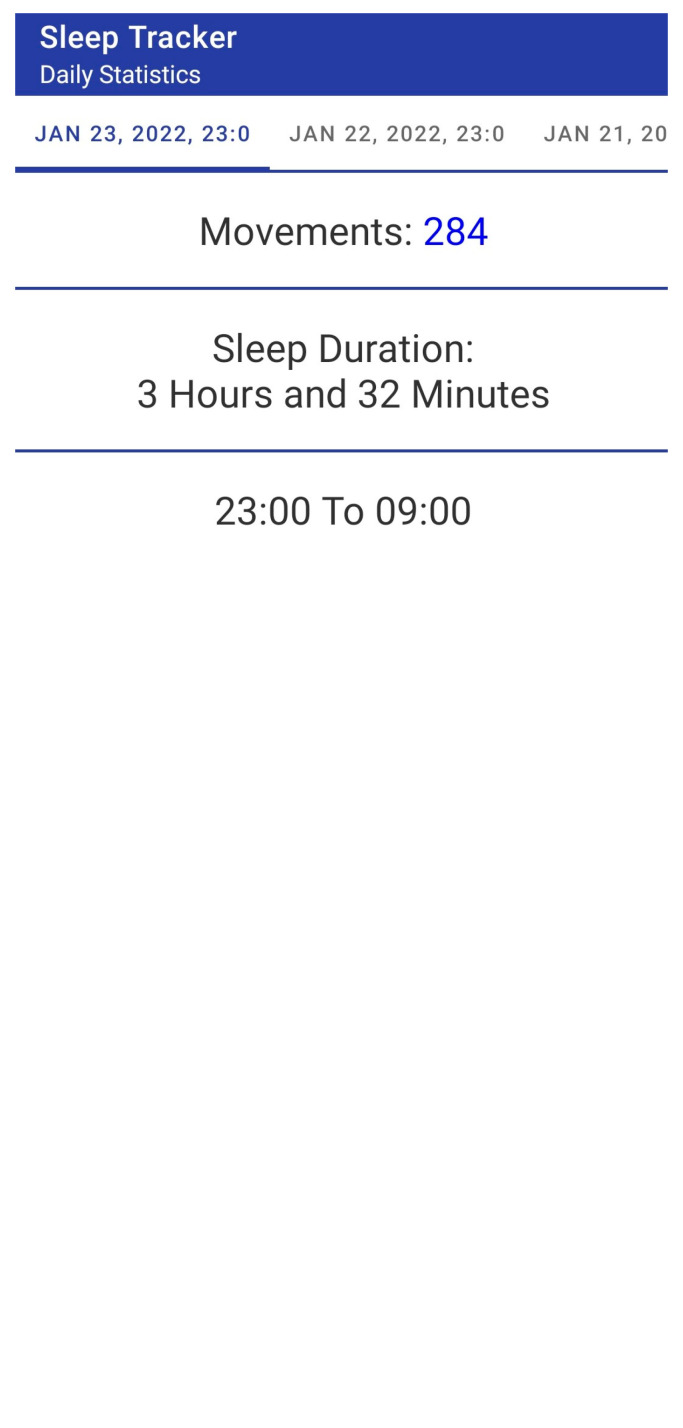
Daily Statistics.

**Figure A7:**
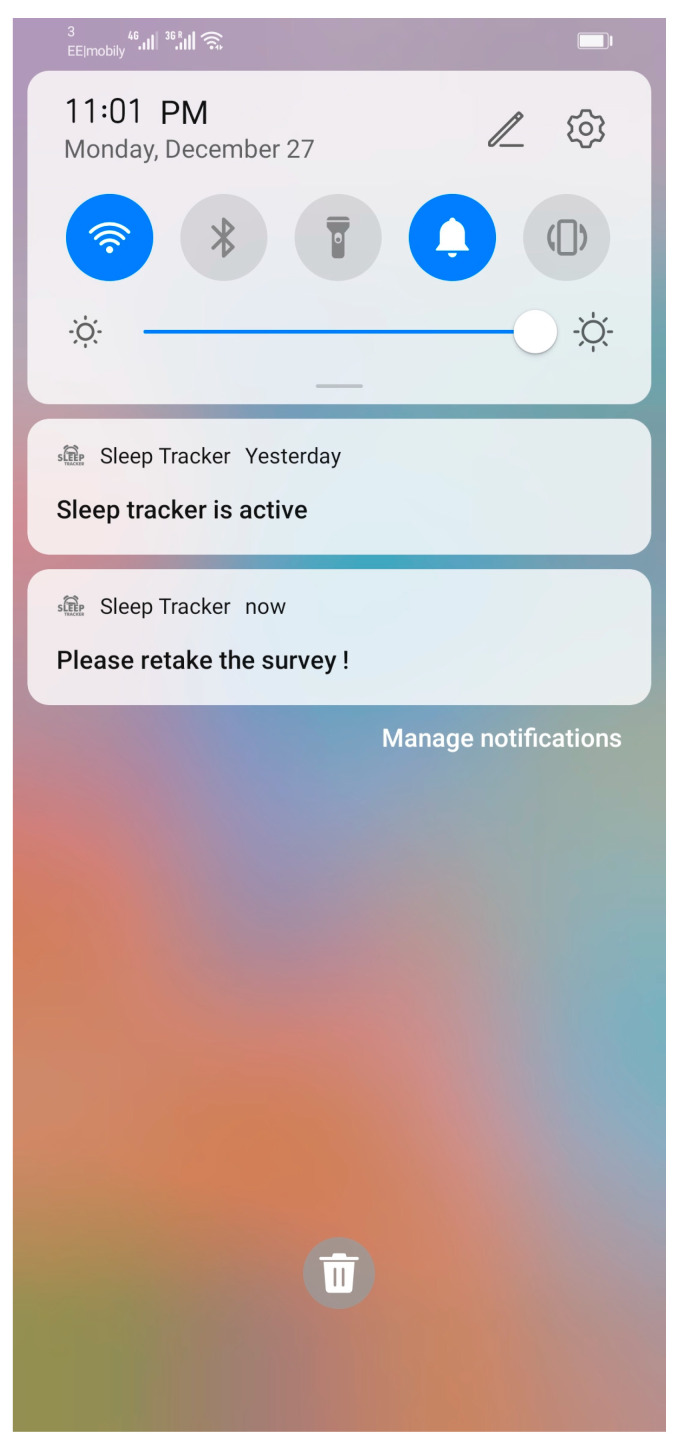

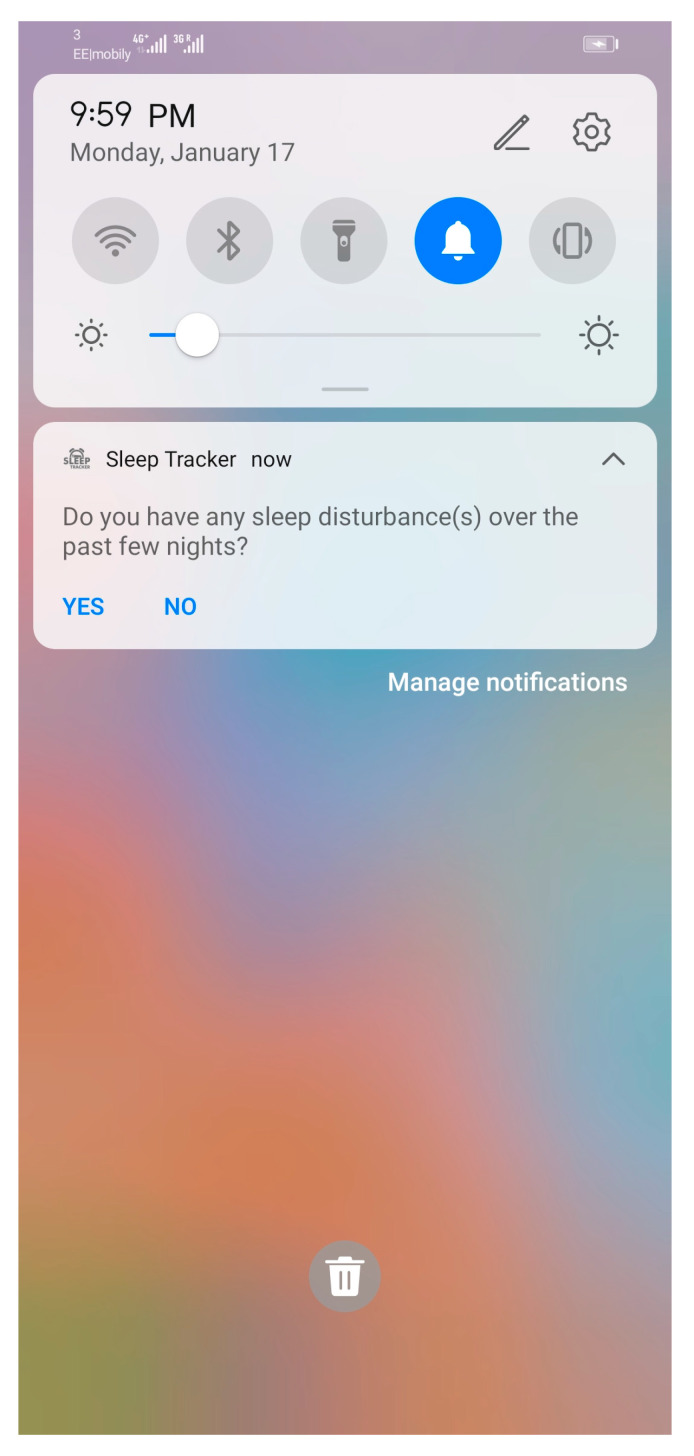
Notifications during the study period if sleep disturbances were detected.

## Data Availability

Data are contained within the article.

## Appendix B

**Table A1:** Data sample collected from users under two conditions using the SleepTracker app, juxtaposed with survey scores for comparison.

User(s)	Condition 1 Screen on/off Events Three Times per Night and for Five Consecutive Days	Condition 2 Phone Movements Three Times per Night and for Five Consecutive Days	Score SCI 1	Score SCI 2	Score SCI 3	Score SCI 4	PHQ-9 1	PHQ-9 2	PHQ-9 3	PHQ-9 4	GAD-7 1	GAD-7 2	GAD-7 3	GAD-7 4
User 1	3	1	6.88	6.25	6.25		6	2	1		5	3	2	
User 2	0	3	6.88	8.75	8.13		0	0	1		2	0	0	
User 3	1	4	1.56		1.56		20		20		17		15	
User 4	0	1	9.69	9.69	9.69		0	0	0		0	0	0	
User 5	0	1	10.00	9.69	9.69		0	0	0		0	0	0	
User 6	6	7	7.81		9.06		1		0		3		2	
User 7	3	4	7.81		9.06		6		6		6		2	
User 8	3	9	6.56	6.56	6.88	6.56	2	3	6	5	3	6	5	5
User 9	0	3	5.63		3.13		10		23		5		9	
User 10	4	3	4.69		4.69		11		7		5		11	
User 11	5	10	8.75	9.06	9.06		2	5	0		0	1	0	
